# Association of *ESR1* polymorphism rs2234693 and rs9340799 with postmenopausal osteoporosis in a Chinese population

**DOI:** 10.1186/s12891-020-03359-2

**Published:** 2020-06-03

**Authors:** Jin Shu, Junling Li, Yujuan Fu, Xuelian Hui, Yani Jin, Mengjie Chen, Xin Zheng, Yaowu Shi

**Affiliations:** Department of Gynecology, Xi’an Hospital of Traditional Chinese Medicine, 69 Fengcheng 8 Road, Xi’an, 710001 China

**Keywords:** Postmenopausal osteoporosis, *ESR1*, Polymorphism, Meta-analysis, Genotype

## Abstract

**Background:**

Postmenopausal osteoporosis (PMO) is the most common type of primary osteoporosis. *ESR1* polymorphism rs2234693 and rs9340799 has been widely studied as a candidate gene associated with PMO, however, the findings were inconclusive. The present study aims to explore the relationship of *ESR1* polymorphism rs2234693 and rs9340799 with PMO risk in a Chinese Han population.

**Methods:**

PMO patients and healthy controls were recruited from gynecology department. DNA of all participants were extracted from the peripheral blood samples and genotyped by Mass Array method. A meta-analysis of case control studies was also conducted to further elucidate the relationship of polymorphism with PMO.

**Results:**

Our results revealed that there were no associations of rs2234693 with PMO. However, GG genotype of rs9340799 was associated with a higher risk of PMO (OR = 1.51, 95%CI:1.08–4.34, *p* = 0.03), even adjusting for risk factors (OR = 1.83, 95%CI: 1.12–5.04, *p* = 0.04). Logistic regression analysis showed that dominant model was associated with a higher risk of PMO (OR = 2.07, 95%CI: 1.02–5.16, *p* = 0.02) after correcting the risk factors (OR = 2.14, 95%CI:1.12–5.64, *p* = 0.04); In addition, the Meta-analysis results revealed that both two polymorphisms were not associated with PMO.

**Conclusions:**

In conclusion, *ESR1* polymorphism rs9340799 was associated with PMO. However, well designed studies with larger sample sizes are required to further elucidate the associations.

## Background

Postmenopausal osteoporosis (PMO) caused by estrogen deficiency, is the most common type of primary osteoporosis which affects more than 40% of postmenopausal women [1–3]. As a typical senile disorder with decreasing bone-mineral density (BMD) and microstructural abnormality, PMO may lead to an increased risk for nonstress fractures [4]. Although, PMO is close related to estrogen levels following menopause, many factors, such as genetic elements had play important roles in the risk of osteoporosis [5, 6]. Among these factors, genetics are found to play a pivotal role in the occurrence of osteoporosis and have received highly attention [6]. Twin studies in adult Caucasian woman revealed that the heritability of BMD might be between 50 and 85% [7]. Genome-wide association study also reported that almost 400 single nucleotide polymorphisms (SNPs) distributed in more than 150 different loci, were associated with low BMD and osteoporosis [8].

Estrogen activity is modulated through estrogen receptor α (ER-α) and β (ER-β) which are encoded by *ESR1* on chromosome 6q25.1 and *ESR2* on chromosome 14q23.2 respectively [9, 10]. Both ER-α and ER-β isoforms are expressed in osteoblasts, osteoclasts, and bone marrow stromal cells [11, 12]. However, *ESR1* is the major mediator of estrogen action in bone and has been widely studied as a candidate gene associated with PMO [13]. Two polymorphisms rs2234693 and rs9340799 had been reported to close related with PMO, however, these findings were inconclusive [14–16]. Mondockova et al. had reported that the rs9340799 was significantly associated with BMD at the femoral neck [17], whereas, another study by tanriover et al. had showed that no relationship of the two genes with PMO [18]. In addition, another study by Tang et al. in a meta-analysis showed that the *ESR1* rs2234693 T allele may increase the risk of hip fracture, but the rs9340799 polymorphism was not associated with hip fracture [19]. Thus, to draw a more precise association of *ESR1* polymorphism (rs2234693 and rs9340799) with the risk of PMO, we sought to assess the impact of *ESR1* polymorphism with PMO and determine a possible association in postmenopausal Chinese women in a case-control study.

## Methods

A total of 380 unrelated postmenopausal women over 45 years old were recruited from the outpatient of Xi’an Hospital of Traditional Chinese Medicine. All the subjects should menopause at least one year before enrollment. BMD was measured by DAX (GE company) and osteoporosis was diagnosed under World Health Organization criteria. Briefly, a T-score above − 1 standard deviation (SD) was considered normal and below − 2.5 SD as osteoporosis. All participants were divided into healthy group and osteoporosis group according to BMD results. Individuals with serious illness and those who accepted hormone replacement therapy (HRT) or drugs (bisphosphonates, steroids, thyroid hormones or GnRH analogs et al.) which may affect bone mass were excluded. Information about risk factors such as body mass index (BMI), menarche, menopause, family and personal history of fractur, smoking habits and alcohol habits were also collected. This study was approved by the Ethical Committee of Xi’an Hospital of Traditional Chinese Medicine and all the researches were performed in accordance with the Declaration of Helsinki.

### Genotyping

Fasting blood samples of each participants were collected and stored at − 80 °C. A commercial kit (Qiagen, Hilden, Germany) was used to extracted the genomic DNA and the genetic polymorphism was identified by the Agena Mass ARRAY system (Agena/Sequenom Inc.) followed the manufacturer’s manual.

### Meta-analysis

Electronic databases including PubMed, ISI Web of Science, National Knowledge Infrastructure (CNKI), and Wanfang Data were searched. MeSH and title/abstract were used for all eligible studies search. Studies included in our meta-analysis need to satisfy the following criteria: (1) studies that conducted in human subjects; (2) sufficient data provided for calculating the crude odds ratios (ORs) and 95% confidence intervals (95% CIs). Correspondingly, the exclusion criteria were those studies without detailed genotype data or reported with overlapping data.

### Statistical analysis

All statistical analyses were performed by SPSS (version 18.0, SPSS Inc., Chicago, USA). Hardy–Weinberg equilibrium (HWE) was examined using the χ2 test. Demographics variables and genotype frequencies between groups were evaluated by student’s t test (for continuous variables) or Chi-squared test (for categorical variables). In addition, multiple logistic regression analyses were also performed to assess the association between osteoporosis as an outcome and risk factors including the *ESR1* polymorphism. Confounding factors were also calculated, including age, gender, BMI, smoking status and drinking habit.

In Meta-analysis, STATA (version 12.0) was employed to make a pooled analysis. Heterogeneity was evaluated by I^2^ statistic. If I^2^ < 50%, the fixed effect model was used, otherwise, the random-effect model was adopted to calculate the pooled ORs. Pooled ORs and 95% CIs were calculated under the following genetic models: (1) allele, (2) recessive, (3) homozygous, (4) heterozygous, and (5) dominant. Publication bias was assessed by Begg’s funnel plots. If *P*-value < 0.05 was considerate to be with significant difference.

## Results

### Characteristics of the study participants

A total of 230 patients and 150 control participants were recruited for this study. All patients were Chinese with confirmed DXA for diagnosis of PMO. There was significant difference of BMD of total hip T-score between patients and control group (*p* < 0.01). No deviation from the HWE was observed (HWE = 0.97) in the control groups. In addition, no significant difference of smoking and drinking ratio were observed between patients and control group. The demographic and clinical characteristics of the participants were shown in Table 1.

**Table 1:** The basic characteristic of the participants

	Control	PMO	P*
Age	54.62 ± 4.43	57.32 ± 6.62	0.06
BMI	21.26 ± 1.25	24.5 ± 3.21	0.04
Age of menopause	47.32 ± 5.69	49.52 ± 7.232	0.08
Years since menopause	5.21 ± 2.14	6.32 ± 1.98	0.22
Age of menarche	12.65 ± 1.21	12.31 ± 1.72	0.12
Total hip T-score	−0.74 ± 1.16	−3.34 ± 1.01	< 0.001
Diabetes(n/%)	15 (10%)	36 (15.6%)	0.08
Hypertension(n/%)	26 (17.1%)	45 (19.5%)	0.09
Smoking(n/%)	2 (1.1%)	2 (0.9%)	0.13
Drinking(n/%)	2 (1.5%)	3 (1.2%)	0.11
Physical activity			0.17
Low level	35 (23.3%)	56 (24.3%)	
Moderate level	72 (48%)	112 (48.7%)	
High level	43 (28.7%)	62 (27%)	

Note: *P* value of the independent sample t-test

### Genetics association analysis

Genotype distribution of *ESR1* gene polymorphisms (rs2234693 and rs9340799) are showed in Table 2. No significant differences were observed between patients and controls in the distribution of TT, TC, and CC genotypes of rs2234693 (*p* = 0.29). Whereas, there was a significant difference in the distribution of AA, AG and GG genotypes of rs9340799(*p* = 0.043).

**Table 2:** The genotype of rs2234693 and rs9340799 polymorphism in PMO and control group. Significant associations are marked in bold

	Control	PMO	P
rs2234693 T > C			
TT	59	79	0.29
TC	70	105	
CC	21	46	
T	188	263	0.13
C	112	197	
rs9340799			
AA	83	116	**0.043**
AG	52	103	
GG	15	11	
A	218	335	0.96
G	82	125	

Note: P value of the independent sample t-test

To further elucidated the relationship of the polymorphism with PMO, a logistic regression analysis was also performed. We found that patients with GG allele of rs9340799 were significantly correlated with PMO morbidity (OR = 1.51, 95%CI:1.08–4.34, *p* = 0.03). After adjusting for age, BMI, smoking and drinking habits, there was still significant association of GG genotype (OR = 1.83, 95%CI: 1.12–5.04, *p* = 0.04) with PMO (Table 3), whereas no significant difference was observed under the AG genotype (*p* > 0.05); In addition, no significant association was observed in rs2234693 with PMO.

**Table 3:** The logistic regression analysis of rs2234693 and rs9340799 genotype with the PMO risk

	OR	95%CI	P	OR*	95%CI*	P*
rs2234693						
TT	Reference			Reference		
TC	0.84	0.31–3.52	0.08	0.86	0.421–3.91	0.08
CC	1.21	0.47–3.19	0.42	1.35	0.58–4.22	0.57
rs9340799						
AA	Reference			Reference		
AG	1.15	0.31–3.52	0.25	1.3	0.51–4.02	0.28
GG	1.51	1.08–4.34	**0.03**	1.83	1.12–5.04	**0.04**

Note: P value of the Chi-squared test; *Adjusting for age, BMI, smoking and drinking habit. Significant associations are marked in bold

We also found a significant association of rs9340799 with PMO under the dominant model (OR = 2.07, 95%CI: 1.02–5.16, *p* = 0.02) even after adjusting for the risk factors (OR = 2.14, 95%CI:1.12–5.64, *p* = 0.04). Nevertheless, no significant association of rs2234693 polymorphism with PMO was observed under dominant and recessive genetic model (Table 4).

**Table 4:** The logistic regression analysis of the dominant model and recessive model with the risk of PMO

	OR	95%CI	P	OR*	95%CI*	P*
rs2234693						
Dominant model	1.14	0.59–1.83	0.37	1.26	0.61–2.62	0.54
Recessive model	1.28	0.68–2.55	0.41	1.50	0.81–3.56	0.45
rs9340799						
Dominant model	2.07	1.02–5.16	**0.02**	2.14	1.12–5.64	**0.04**
Recessive model	1.76	0.71–4.04	0.22	1.91	0.89–4.87	0.31

Note: P value of the regression analysis; *Adjusting for age, BMI, smoking and drinking habit. Significant associations are marked in bold

### Meta-analysis results

**Fig. 1:**
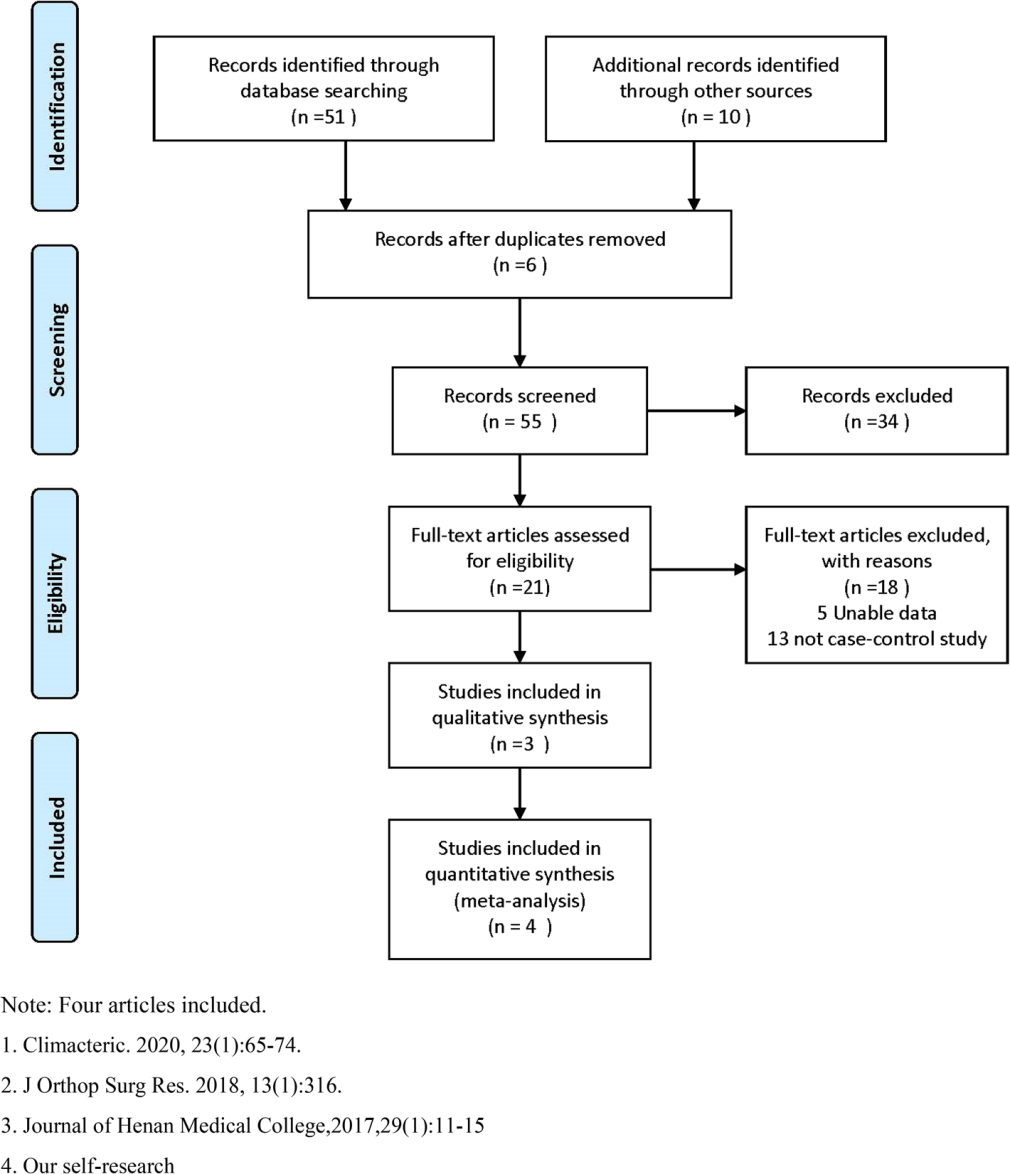
Flow sheet summarizing study identification and selection. Note: Four articles included. 1. Climacteric. 2020, 23(1):65–74. 2. J Orthop Surg Res. 2018, 13(1):316. 3. Journal of Henan Medical College,2017,29(1):11–15. 4. Our self-research

**Fig. 2:**
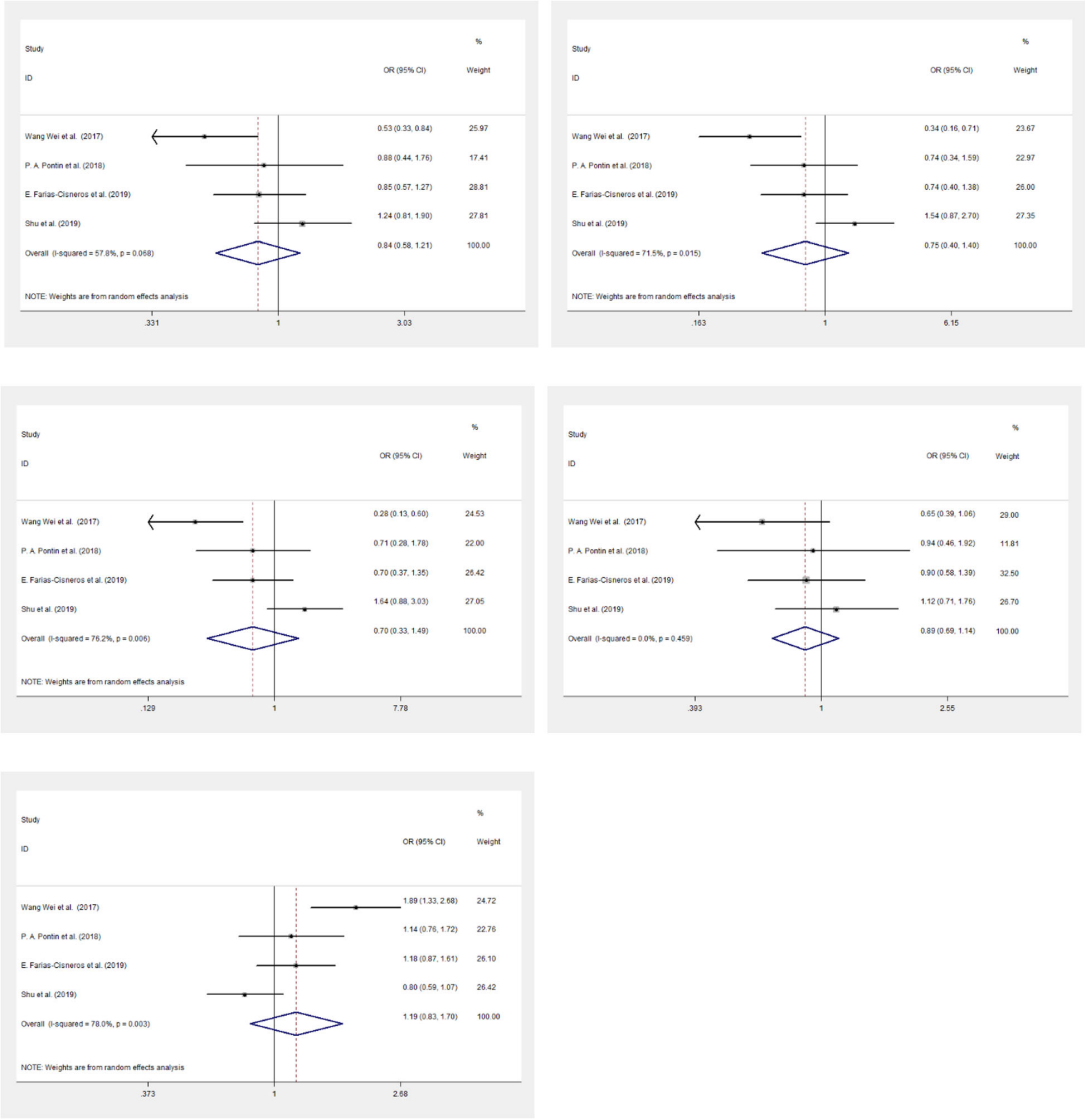
Forest plots of rs2234693 polymorphism under five genetic models. A is the dominant model; B is the recessive model; C is the homozygote model; D is heterozygote model; E is the allelice model

**Fig. 3:**
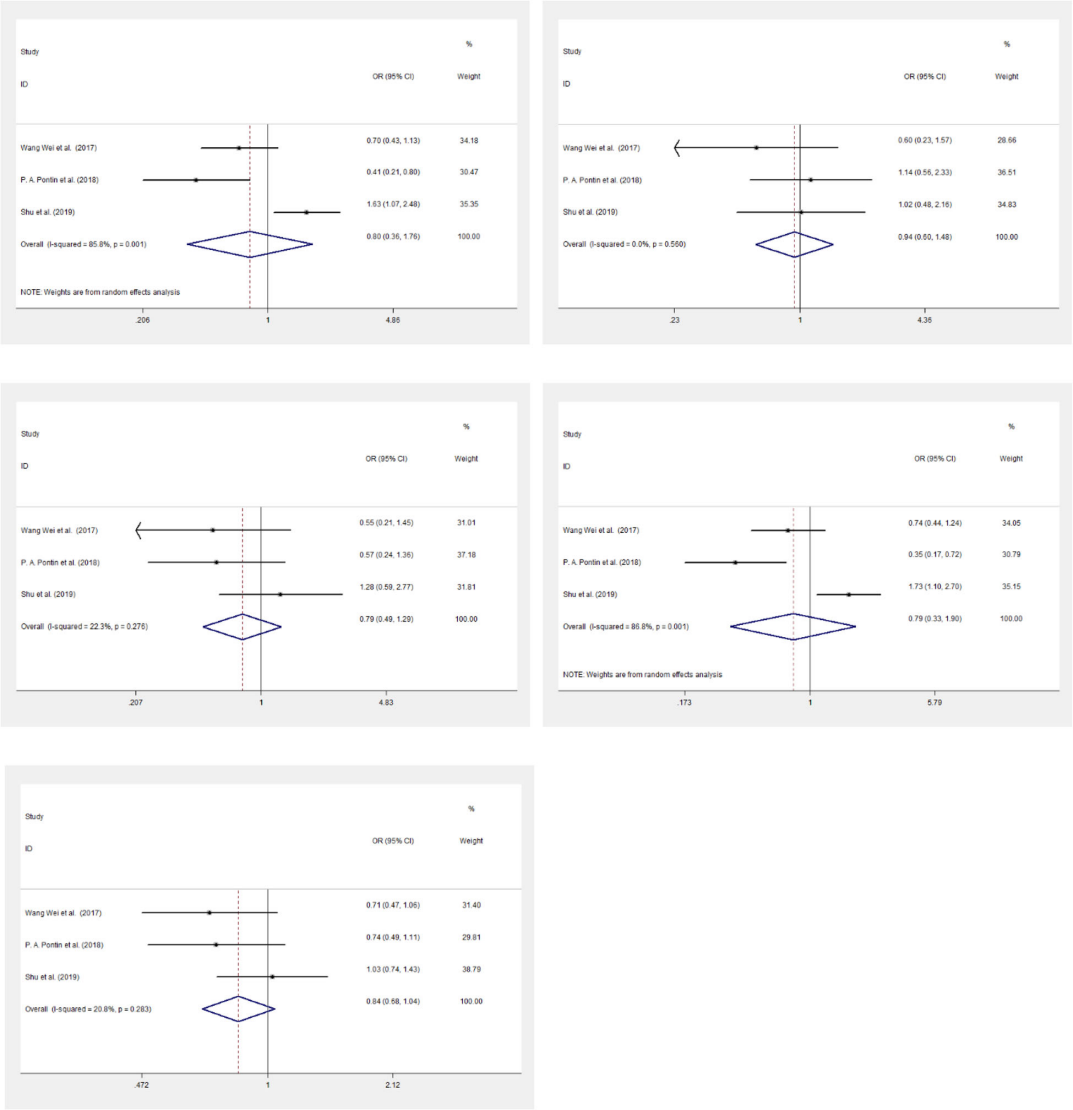
Forest plots of rs9340799 polymorphism under five genetic models. A is the dominant model; B is the recessive model; C is the homozygote model; D is heterozygote model; E is the allelice model

**Fig. 4:**
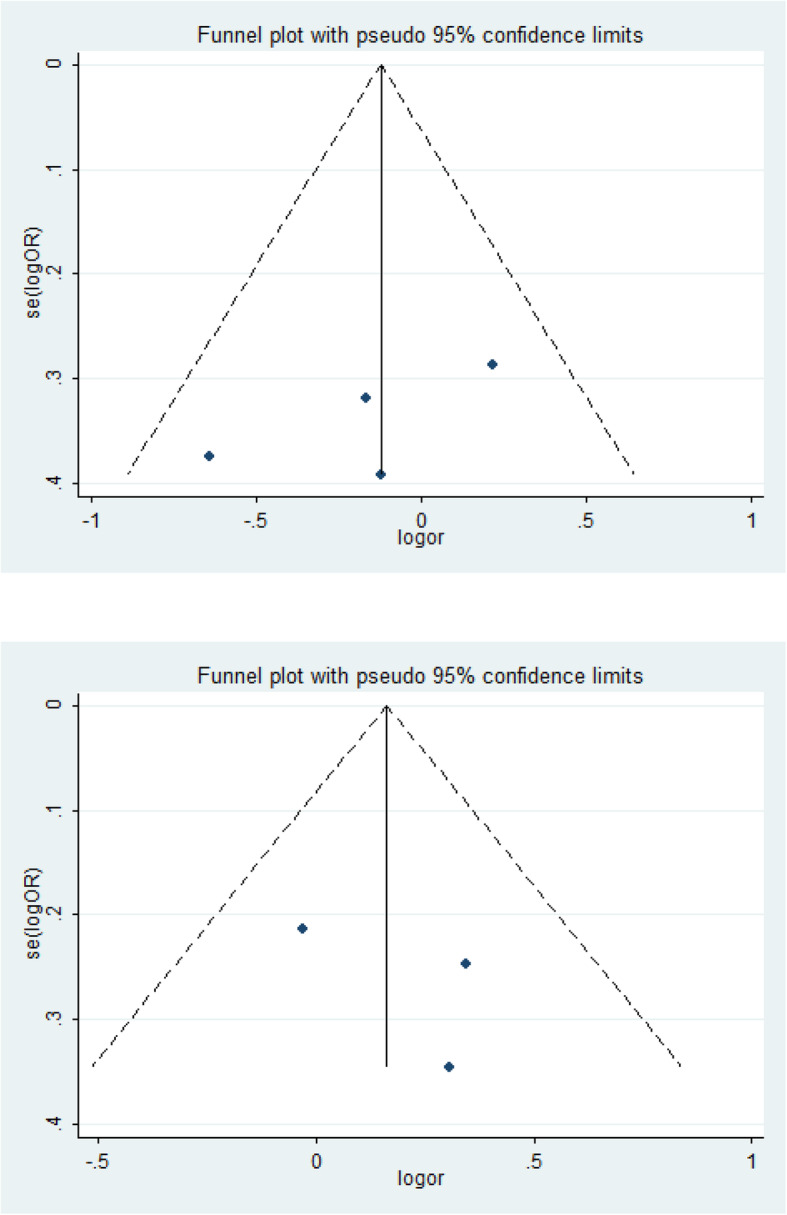
Begg’s funnel plot for publication bias analysis (dominant model)

A meta-analysis was also employed to further explore the relationship of *ESR1* polymorphism with PMO. A total of 4 articles (including our results) were enrolled in the analysis (Fig. 1). The study characteristics were listed in Table 5. The random effect model was employed under the dominant, recessive, homozygous, heterozygous and allelic model for rs2234693. Whereas, for rs9340799, only dominant model was found with heterogeneity, thus the fixed model was used in the other four genetic models. The meta-analysis results showed that there were no association of rs2234693 and rs9340799 with PMO (*p* > 0.05). (Fig. 2 and Fig. 3). Publication bias evaluated by using Begg’s test show that no significant publication bias exists (p > 0.05) (Fig. 4).

**Table 5:** Characteristics and genotype of the studies included in this meta-analysis

Author	Year	Age		MPO rs2234693/(rs9340799)					Control rs2234693/(rs9340799)					Method
		MPO	control	TT/(AA)	TC/(AG)	CC/(GG)	T/(A)	C/(G)	TT/(AA)	TC/(AG)	CC/(GG)	T/(A)	C/(G)	
Wang et al. [20]	2017	62. 81 ± 5. 78	64. 21 ± 9. 22	78/99	53/36	11/7	209/234	75/50	59/93	62/46	30/12	180/232	122/70	PCR-LDR
Pontin et al. [16]	2018	> 40	> 40	22/33	56/39	14/20	100/105	84/79	20/17	54/57	18/18	94/91	90/93	PCR-RFLP
Cisneros et al. [21]	2019	66 ± 11.2	58.1 ± 8.4	91/(N/A)	70/(N/A)	19/(N/A)	252/(N/A)	108/(N/A)	91/(N/A)	78/(N/A)	27/(N/A)	260/(N/A)	132/(N/A)	TaqMan
Shu et al.	2019	57.32 ± 6.62	54.62 ± 4.43	79/115	105/96	46/19	263/326	197/120	59/93	70/45	21/12	188/221	112/79	Mass-Array

## Discussion

Osteoporosis is a multifactorial disease, characterized by loss of tissue microarchitecture and low BMD. It has been estimated that 30% of women and 12% of men were affected by osteoporosis [8, 22]. The most important adverse health outcome of osteoporosis is bone fractures. Women at postmenopausal stage are faced to extremely high risk of osteoporosis [23–25]. Several candidate genes, such as *ESR1*, the major mediator of estrogen action in bone, have been reported to be associated with BMD and osteoporosis [26–28]. Ioannidis et al. had revealed that *ESR1* is a susceptibility gene for fractures [29]. However, the polymorphism of *ESR1* with PMO was still inconclusive.

Studies to elucidate the *ESR1* genetic contributions to PMO have continued for several decades. To a much lesser extent, the association between BMD and a polymorphism in the promoter region of *ESR1*, characterized by a variable number of studies and still not come to a unified conclusion. Mondockova et al. had found that rs9340799 polymorphism may contribute to decreased BMD in postmenopausal women in southern Slovakia [14]. Nevertheless, Wang et al. had showed that rs2234693 polymorphism but not rs9340799 was associated with PMO [23]. And Kurt et al. showed that both rs9340799 and rs2234693 polymorphism were contribute to the determination of bone mineral density in Turkish postmenopausal women [30]. In our study, we had found that the GG genotype and the dominant genetic model of rs9340799 were susceptible to PMO, whereas, no relationship was found in rs2234693. These results were partly in accordance with former studies.

Although our case-control study had got the positive conclusions of rs9340799 polymorphism with PMO, these results should be treated with caution. A meta-analysis was also conducted to further elucidate the relationship of disease and polymorphism. Our meta-analysis of pooled analysis had showed that either homozygote, heterozygote, recessive, allelic models or dominant of rs9340799 and rs2234693 were the risk factor of PMO. These may be attributed to a small sample size, different ethnic background and different examine methods. These data need to be replicated in a larger cohort, and functional studies will be necessary to investigate whether and how *ESR1* gene polymorphism involved in the pathogenesis of PMO.

The rs2234693 and rs9340799 polymorphic sites are located in the promoter region of the first intron of *ESR1* gene, and so far, their functional consequences are unknown [21]. Although case-control study had partly revealed the relationship of the polymorphism, its mechanism is still not clear. We speculate that introns may contain regulatory elements, and the mutation may cause methylation and finally influence the effect of *ESR1*.

The results of our study may help in identifying patients with potential PMO risk; however, several limitations should not be ignored. Firstly, as the quantity of the patients were not large enough, thus give rise to failure to achieve statistical significance of rs2234693. In addition, there was significant difference under BMI in the baseline characteristic, this may cause the bias to the results. Secondly, all participants enrolled were recruited from hospital which might result in potential selection bias. Thirdly, some potential confounding factors which may overestimate or underestimate the effect of gene polymorphism. Eventually, the meta-analysis was only enrolled 4 relative studies, this may give rise to publication bias and finally influence the overall results.

## Conclusion

We had reported a significantly correlation of *ESR1* genotype distribution with PMO in a Chinese Han population. This result was partly in accordance with the meat-analysis results. However, this is only a preliminary conclusion, a larger cohort study and functional studies will be necessary to investigate whether and how the polymorphism might involve in the pathogenesis of PMO.

## Data Availability

The datasets generated and/or analyzed during the current study are not publicly available due to potential for individual and organizational privacy to be compromised. Reasonable requests for parts of the data will be considered by the corresponding author.

## Abbreviations

PMO: Postmenopausal osteoporosis
BMD: Bone-mineral density
DXA: Dual energy x-ray absorptiometry
HRT: Hormone replacement therapy
BMI: Body mass index
HWE: Hardy–Weinberg equilibrium
95% CI: 95% confidence interval
SNP: Single nucleotide polymorphisms
